# Role of hypertonic saline and mannitol in the management of raised intracranial pressure in children: A randomized comparative study

**DOI:** 10.4103/1817-1745.66673

**Published:** 2010

**Authors:** Piyush Upadhyay, V. N. Tripathi, R. P. Singh, D. Sachan

**Affiliations:** Pediatric Intensive Care Unit, Department of Pediatrics, GSVM Medical College, Kanpur (U.P.), India

**Keywords:** Hypertonic saline, mannitol, raised intracranial pressure

## Abstract

**Objective:**

To compare the efficacy and side effects of 3% hypertonic saline and mannitol in the management of raised intracranial pressure in children.

**Design:**

Prospective randomized study.

**Setting:**

Pediatric intensive care unit (PICU) in a tertiary care hospital.

**Subject:**

200 patients with raised intracranial pressure.

**Materials and Methods:**

Patients were randomized into two statistically comparable groups; Group A (n = 98) was treated with mannitol while Group B (n = 100) was treated with 3% hypertonic saline. Group C (n = 2) included those members of Group A in whom serum osmolality ≥320 mosmol/kg and were then treated with 3% hypertonic saline. Both Drugs were given at a loading dose of 5 ml/kg stat followed by 2 ml/kg in every 6 h(both have same osmolarity) for two days in their respective groups. Besides monitoring, blood pressure (NIBP), mean arterial pressure (pre and post 30 min of drug), serum sodium, chloride and osmolality were measured. Intracranial pressure was assessed indirectly by measuring mean arterial ressure “MAP”. Student paired ‘t’ test was applied.

**Results:**

Decrease in MAP was highly significant (*P*<0.001) at 0 h in males 0,6 h in females, and moderately significant at 12,36 h in females and significant(*P*<0.05) at 6,24,42 h in males of Group B. Decrease in coma hours was a highly significant finding (*P*<0.001) in Group B. In Group B, serum sodium and chloride increased significantly but remained within acceptable limits. There was no difference in osmolality and mortality (fisher Z).

**Conclusion:**

Mannitol has several side effects, 3% hypertonic saline is a safe and effective alternative in managing cerebral edema.

## Introduction

Hyperosmolar treatment is one of the important methods for treating cerebral edema, and has been employed since early 1960. Urea, glycerol and mannitol were used for the treatment of this condition in the early years, but urea and glycerol were soon abandoned because of low efficacy. Mannitol is still used extensively. Side effects such as rebound effect, serum electrolyte imbalance and hypovolemia have led to the continued search for other osmotically active agents. One of them is hypertonic saline.

### Aims and objectives

To study the efficacy of hypertonic saline in the treatment of raised intracranial pressure in children in comparison to mannitol.

To study the side effects (if any) of hypertonic saline treatment in the management of raised intracranial pressure.

## Materials and Methods

- **Study design:** Prospective randomized comparative study
- **Setting:** Pediatric intensive care unit (PICU)
- **Data analysis:** Continuous data was analyzed by using Student unpaired ‘t’ test
- **Inclusion criteria:** 200 Children aged 2 to 18 years, admitted to the emergency Department of Pediatrics between Jan 15th, 2007 and May 31^st^, 2008 with clinical symptoms and signs of raised intracranial pressure.

- **Exclusion criteria**
  Patients with:
  - compromised renal function (increased Serum creatinine)
  - hepatic encephalopathy
  - serum Na+ (>150 meq/l)
  - diabetic ketoacidosis
  - cerebral malaria

### Groups and method of randomization

Group A (those admitted on Wednesday, Thursday, Friday, *n* = 98): treated with mannitol

Group B (those admitted on Monday, Tuesday, Saturday, *n*=100): treated with 3% hypertonic saline.

Group C (*n* = 2) was later made and Included those members of Group A in whom S. osmolality increased to more than 320 mOsm / kg and were then treated with 3% hypertonic saline.

### Intervention protocol

Loading dose(5ml/kg) was followed by maintenance dose (2 ml/kg) in every 6 h in both groups for two days (osmolarity of mannitol and 3% hypertonic saline are almost same i.e. 1100 mOsm/l and 1098 mOsm/l, respectively).

### Measurements taken

**Blood pressure:** By NIBP, at admission and then in every 6 h, both pre and post drug (30 min of 3% hypertonic saline or mannitol therapy).

**S. creatinine:** Before starting treatment and after 48 h.

**Blood urea nitrogen:** Before starting treatment and after 48 h.

**Serum electrolytes:** Before starting treatment and then in every 12 h.

**ABG:** Before starting treatment, and after 12, 24, 36 and 48 h.

### CT scan (or MRI when needed) of head

Comparison of average reduction of mean arterial pressure (pre and post drug) at definite time (6h) intervals was done to indirectly assess reduction in intracranial pressure.

Mean arterial pressure indirectly represents intracranial pressure

(Cerebral perfusion pressure (CPP) = mean arterial pressure – intra cranial pressure (ICP) i.e. MAP = CPP + ICP (where CPP remains constants)

Formula applied: MAP α ICP

## Results

- 3% hypertonic saline was more efficacious than mannitol in the initial 12 h and equally or more efficacious than mannitol therapy later.
- Decrease in coma hours was a significant additional finding in the group treated with 3% hypertonic saline (Group B).
- Change in blood biochemistry was within acceptable limits in Group B. [Tables 1–5]

**Table 1:** Age and Sex wise distribution of trial groups

Groups	Mean age (mths)	Female		Male	
		No. of cases(n)	%	No. of cases(n)	%
A	67.9	40	40	58	58
B	68.4	39	39	61	61
C	70.5	1	50	1	50
	Total	80	40	120	60

Age and sex distribution between A & B trial groups was comparable

**Table 2:** Etiological distribution of trial group

Etiology	Group A	Group B	Group C
Meningoencephalitis	73 (74.48)	75 (75)	2 (100)
Neurocysticercosis (Anoxic Encephalopathy)	12 (12.24)	8 (8)	
Head trauma	6 (6.12)	8 (8)	
Space occupying lesion	5 (5.10)	5 (5)	
Global Infarction	2 (2.04)	4 (4)	
Total (n)%	98	100 (100)	2

Thus etiological distribution in group A&B were found to be comparable, Figures in the parentheses are in percentage

**Table 3:** Comparison of various parameters between group A & B

Parameter (X±SD)	Group A	Group B	Group C	‘t’ value	‘*P*’ value	Inference
S. Osm (mosm/kg)	310±4.3 (285-319)	308±5.6 (288-322)	330±8.4 (324-336)	1.41	>0.05	Not Significant
S.Na (meq/l)	128±2.4 (123-130)	136±6.2 (122-153)	138±8.4 (132-144)	6.20	<0.001	Highly Significant
Duration of coma (hrs)	98.6±21.25 (28-138)	77.50±13.05 (24-144)	123±18.3 (110-136)	8.40	<0.001	Highly Significant
S.Chloride (mmol/l)	96.0±1.3 (85-99)	102±1.6 97-106)	100±2 (98-104)	3.00	<0.05	Significant
Mortality	5	4	0	Z=0.34 Fisher Z	>0.05	Not Significant

**Table 4:** Relationship between group A and group B male subjects

Hrs	Group A (X±SD)	Group B (X±SD)	‘t’ value	‘*P*’ value	Significance of B
0	7±3.25	11.5±4.48	6.33	<0.001	Highly Significant
6	7±2.58	8.2±3.52	2.14	<0.05	Significant
12	5.7±1.62	5.4±1.97	0.97	>0.05	Not Significant
18	6.6±2.89	6.1±2.6	1.0	>0.05	Not Significant
24	6.2±2.56	5±2.51	2.60	<0.05	Significant
30	4.7±2.14	4±2.59	1.62	>0.05	Not Significant
36	3.3±2.58	3.6±2.25	0.68	>0.05	Not Significant
42	2±2.19	2.8±2.45	2.00	<0.05	Significant
48	2±1.8	1.9±1.14	0.37	>0.05	Not Significant

X=mean of differnce between pre and post drug mean map at definite time intervals

**Table 5:** Relationship between group A and group B female subjects

Hrs	Group A) (X±SD)	Group B (X±SD)	‘t’ value	‘*P*’ value	Significance of B
0	7±1.88	8.2±1.74	4.80	<0.001	Highly Significant
6	5.8±1.16	4.4±2.02	3.15	<0.001	Highly Significant
12	5.0±1.47	6.1±1.80	2.9	<0.01	Moderate Significant
18	2.7±1.18	3.3±1.99	1.66	>0.05	Not Significant
24	3.4±1.91	4.0±2.06	1.36	>0.05	Not Significant
30	4.6±1.17	3.38±1.06	3.20	<0.01	Moderate Significant
36	4.2±1.20	4.35±1.27	0.55	>0.05	Not Significant
42	2.6±1.73	3.0±1.60	1.35	>0.05	Not Significant
48	1.6±1.33	1.30±0.56	1.36	>0.05	Not Significant

X=mean of differnce between pre and post drug mean map at definite time intervals

## Discussion

For the first time, in 1919, Weed and McKibben[2] reported in animal models that HS results in a change in the brain volume. However, HS failed to attract the interest and field of application it deserved. In the early 1980’s, its positive effects were shown in patients with hemorrhagic shock.[3] Later, it was employed in animal models with traumatic cerebral edema and shown to be superior than mannitol in reducing intracranial pressure (ICP) and fluid content of brain.[4,5] These experimental findings gave encouragement for application to patients with cerebral edema of traumatic origin. Worthley, *et al*.[7] demonstrated reduction in ICP and increase in systemic perfusion with a 30% saline given as a single bolus in two traumatic mannitol resistant patients. Like-wise, in a few uncontrolled studies HS at 3-23.4% has been shown to reduce the ICP after head trauma.[8,9]

During the period in which mannitol was used intensively, maintenance of serum osmolarity below 320 mOsm/l was recommended because of complications of acute tubular necrosis (ATN) and renal failure. However, it was later understoodthat this complication developed as a result of dehydration and hypovolemia.[10] Children have been found to tolerate well the rather high serum osmolarity (365 mOsm/l) due to HS.[11,12] Owing to its diuretic effect and the consequent risk of development of hypovolemia, mannitol has a greater risk of being complicated by ATN than HS. Comparative studies with mannitol have also been conducted and published. In a prospective, randomized study, Vialet, *et al*.[13] showed that HS at 7.5% concentration administered as an isovolemic bolus (2 ml/kg) was more effective than 20% mannitol in reducing the ICP in trauma patients. Another prospective study conducted by Horn, *et al*.[14] using 7.5% saline administered as bolus infusion to patients with elevated ICP due to trauma and not responding to the standard treatment showed that it was effective in reducing the ICP and CPP. With the aim of reducing the ICP to below 20 mmHg, Peterson, *et al*.[11] administered a 3% saline infusion to 68 children with trauma who did not respond to standard treatment. They found serum-Na concentrations of 150-170 mEq/l and a serum osmolarity of 300-330 mOsm/l to correlate with better prognosis. Our study is a prospective analysis of one and a half year period with a patient population consisting of children. Our cases and etiologic factors are different from other studies. In our study, the etiologic factors included were infection, hemorrhage, anoxia, and trauma factors. ICP measurement could not be conducted in our study, so treatment was continued considering the serum-Na concentration and osmolarity until clinical improvement was achieved. We have shown better results in Group B and Group C with no significant side effects. The main disadvantage is the fact that in our study direct ICP measurement was not conducted. However, compared with mannitol, the clinical efficacy has also been confirmed by mortality assessment.

Hypertonic saline has been used more frequently in trauma, intracerebral hemorrhage, burn and stroke patients. Our patient group was different in their etiology of brain edema. Rationale of use of mannitol and HS is similar in both traumatic and non traumatic cerebral edema because all cerebral edemas with varied etiologies usually have vasogenic mechanism. In addition, HS is more effective and safer than mannitol. Greater efficacy of HS compared to mannitol can be speculated by different reflection coefficients of these agents.[1] Because the reflection coefficient for Na and Cl was 1.0, such a side effect in saline treatment may not be expected. To our knowledge, there is rarely any report of HS treatment in edema of anoxic, infectious origin in the recognized literature.

In terms of the efficacy and side effect profile of the saline treatment in brain edema, the optimum serum-Na concentration and osmolarity are not known. In a retrospective study Peterson *et al*.,[11] though making no comparative analysis, suggested that prognosis in patients with serum-Na concentration within the range of 150-170 mEq/l and serum osmolarity of 300-340 mOsm/l seems to be better. Also, some studies report an inverse relationship between serum-Na concentration and ICP.[9–11] There was no significant difference in our patients with Na level of 150-160 and 160-170 mEq/l in terms of duration of the comatose state and mortality. As during the hyperosmolar state, to maintain the osmotic balance, idiogenic hyperosmoles are formed within the cells in 72-96 h.[15–18] So after thetermination of HS, serum-Na concentration should be gradually reduced over a period of more than two days.

Potential side effects of hypertonic saline have been reported:[19] Myelinolysis, acute tubular necrosis and renal failure, subdural hematoma or effusion, heart failure, pulmonary edema, hypokalemia, hyperchloremic metabolic acidosis, coagulopathy, intra-vascular hemolysis, and rebound cerebral edema may occur. Myelinolysis occur more frequently if there is a rapid transition from hyponatremia to hypernatremia. For myelinolysis to occur, a daily serum-Na concentration load of 35-40 mEq/l is required.[20] The region most susceptible to myelinolysis is the pontine white matter with visualized MRI and central pontine myelinolysis is manifested clinically as lethargy and quadriplegia/paresia. We have not observed acute flaccid paralysis/plegia after saline treatment. Especially, in patients with significant degrees of cerebral injury, the clinical assessment of this complication could not be detected possibly by the fact that they had blunted mental status.

Renal failure, congestive heart failure, pulmonary edema, hypokalemia and phlebitis were not observed in any of our patients. There was no significant tendency for hemolysis or hemorrhage associated with acute fall in the hematocrit level.

In conclusion, in the treatment of cerebral edema of infectious, anoxic, hemorrhagic and traumatic origin, administration of HS is probably more effective and safer than mannitol. However, to determine just when to initiate treatment, how long to continue treatment, and target serum-Na concentration requires monitoring of the intracranial pressure. Further studies are required to resolve these concerns.
